# Bioactive compounds from *Hypericum humifusum* and *Hypericum perfoliatum*: inhibition potential of polyphenols with acetylcholinesterase and key enzymes linked to type-2 diabetes 

**DOI:** 10.1080/13880209.2016.1270973

**Published:** 2017-02-01

**Authors:** Afef Béjaoui, Issam Ben Salem, Nesrine Rokbeni, Yassine M’rabet, Mohamed Boussaid, Abdennacer Boulila

**Affiliations:** a Laboratory of Plant Biotechnology, National Institute of Applied Sciences and Technology, Tunis, Tunisia;; b Laboratory of Natural Substances, National Institute of Research and Physico-chemical Analysis, Ariana, Tunisia;; c National Centre for Nuclear Science and Technology (CNSTN), Ariana, Tunisia

**Keywords:** Hypericum species, hypericin, hyperforin, antioxidant, enzyme inhibitory, antimicrobial

## Abstract

**Context:** Natural products are reported to have a wide spectrum of pharmacological properties such as antimicrobial, anti-inflammatory and anti-cholinesterase. The genus *Hypericum* (Hypericaceae) is a source of a variety of molecules with different biological activities, notably hypericin and various phenolics.

**Objectives:** The goals of the present work were the determination of total phenolic and flavonoid content, hypericin and hyperforin concentration as well as the evaluation of biological of *Hypericum humifusum* L. (Hhu) and *Hypericum perfoliatum* L. (Hper).

**Materials and methods:** The various extracts of aerial parts were powdered, and then extracted with methanol. Antibacterial activity was performed according to minimum inhibitory concentration (MIC) and minimum bactericidal (MBC) methods against four Gram-positive bacteria, four Gram-negative bacteria and yeast.

**Results:** The results revealed that *H. humifusum*, bear the highest total phenolic and flavonoid content (48–113 mg GAE/g and 8–41 mg RE/g, respectively) as well as hypericin (60–90 mg/g) and hyperforin (8–30 mg/g) concentration. Both species showed significant antioxidant activity as revealed by DPPH, FRAP, ABTS, and metal chelating assays. *H. humifusum* exhibited a strong acetylcholinesterase (3.86–4.57 mg GALAEs/g), α-glucosidase (0.73–2.55 mmol ACEs/g) and α-amylase (3–8 mmol ACEs/g) inhibitory activity. The extract of *H. humifusum* exhibited strong antibacterial activity mainly against *Staphylococcus epidermidis*, *Staphylococus aureus*, and *Enterococcus faecium* (MIC values ranging from 200 to 250 μg/mL). The highest antifungal activity was showed for *H. perfoliatum* extract (MIC value = 250 μg/mL).

**Conclusion:** The data suggest that *H. humifusum* could be used as valuable new natural agents with functional properties for pharmacology industries.

## Introduction

The genus *Hypericum* L. (Hypericaceae) contains 484 species that have been classified into 36 taxonomic sections in all the continents except Antarctica. *Hypericum* species are perennial, herbaceous, or scrubby. There has been increasing interest in the genus *Hypericum*, because it is a source of a variety of molecules with different biological activities (Kizil et al. 2004), notably hypericin and various phenolics. The concentration of these compounds in plant tissues vary among plant parts, phonological stages and origin. These compounds exhibited a wide spectrum of pharmacological properties such as antimicrobial (Agostinis et al. 2002), anti-inflammatory, and anti-cholinesterase (Kamatou et al. 2008; Cardile et al. 2009), anticancer (Butterwerck et al. 2000), antidepressant (Jacobson et al. 2001), antiviral (Cakir et al. 2003), antioxidant (Ali et al. 2011), antifungal and antimalarial (Unal et al. 2008) activities. Benzopyran of xanthones and flovonoid derived from these compounds are known to have antimicrobial activity against various fungi and bacteria (Conforti et al. 2005).

Tunisian flora contains eight *Hypericum* species; *H. perforatum* L., *H. humifusum* L., *H*. *tomentosum* L., *H. perfoliatum* L., *H. triquetrifolium* Turr., *H. ericoides* L., *H. androsaemum* L., and *H. afrum* Desf. (Pottier-Alapetite 1979).

*Hypericum humifusum* L. is a short perennial and diploid species (Matzk et al. 2003) common in the Mediterranean area, and in western, and central Europe (Nogueira et al. 2008). It is an outcrosser, reproduces by seeds, and shows some capacity to reproduce vegetatively through sprouts. Leaves are simple and lanceolate. Flowers, grouped in a terminal corymb, are yellow, veined of red. However, it is widely used in folk medicine for its antiseptic, astringent and antispasmodic effects (Le Floc’h 1983). This species possess a high content of naphtodianthrones, which might be used against viruses and retroviruses, and considerable amounts of phloroglucinol with potential antibacterial and cytotoxic activities (Stojanović et al. 2013).

*Hypericum perfoliatum* L. is a perennial herbaceous species, usually growing in shady places among rocks. It has 20–50 cm height, 15–80 cm stems, 1.5–4.0 cm leaves, ovate to triangular-lanceolate, the upper most sometimes black-glandular-ciliate, usually densely pellucid-dotted, with obscure reticulate venation. The species has oblong, subacute to rounded sepals, which are densely and irregularly dark-glandular denticulate; petals 2–3 times great than calice, with black dotes. Its fruit is a 0.5–0.6 cm capsule, ovate or obovate, with dorsal vittae and lateral vesicles (Çirak 2007; Del Monte et al. 2015; Pottier-Alapetite 1979). *Hypericum perfoliatum* has great pharmaceutical potential (Sarrell et al. 2003), with well documented contents of hypericin (Ayan et al. 2004), phenolics, phloroglucinols (Benkiki et al. 2003) and essential oils (Couladis et al. 2001; Çirak et al. 2007).

*Hypericum perforatum* L. is the most studied taxon (Vinterhalter et al. 2006). It is used for the extraction of several bioactive compounds known for their photodynamic, antidepressive and antiviral activities (Patocka 2003). The other species remain little known with regard to their chemical composition especially for their hypericin and hyperforin content, the most studied molecules from the genus. Both *in vitro* and *in vivo* studies, hypericin significantly inhibited viruses such as HIV (Human immunodeficiency virus) 35, Influenza A, Human cytomegalovirus (HCMV), the DNA-virus, i.e. Herpes simplex (type 1 and type 2) (Westh et al. 2004; Fritz et al. 2007).

In an attempt to broaden our knowledge of chemical composition of Tunisian medicinal flora, we have undertaken an analysis of total phenolic and flavonoid content, concentration in hypericin and hyperforin in *H. humifusum* and *H. perfoliatum* as well as their biological activities including antioxidant, antimicrobial, acetylcholinesterase, α-amylase and α-glucosidase inhibitory effect.

## Materials and methods

### Chemicals and reagents

Folin–Ciocalteu reagent, 1,1-diphenyl-2-picrylhydrazyl (DPPH), 2,4,6 tripyridyl-s-triazine (TPTZ), 6-hydroxy-2,5,7,8-tetramethylchroman-2carboxylic acid (Trolox), iron(III) chloride hexahydrate (FeCL_3_·6H_2_O), Iron(II) chloride tetrahydrate (FeCl_2_·4H_2_O), ferrozine, iron(II) sulfate heptahydrate (FeSO_4_·7H_2_O), potassium iodide (KI), α-amylase, α-glucosidase and acetylcholinesterase were purchased from Sigma-Aldrich (St. Louis, MO). All solvents and reagents used in this study are of higher purity.

### Plant materials

*Hypericum humifusum* L. and *H. perfoliatum* L. were collected from the northern west of the country from the sub-humid bioclimate during summer 2007 from Tunisia. Botanical identification of this species was carried out by Prof. Mohamed Boussaid, biologist (National Institute of Applied Science and Technology, Tunisia). Voucher specimens were deposited at the herbarium of INSAT.

### Extraction

Approximately 10 g of each plant was powdered, and then extracted with 100 mL MeOH under stirring at 200 rpm for 4 days. The solutions were filtered and concentrated under reduced pressure to evaporate the solvent and then dried under nitrogen ﬂow. The residue was redissolved in MeOH and then stored at −20 °C until further analysis.

### Determination of total phenolics

Total phenolics contents were assayed using the Folin–Ciocalteu reagent and gallic acid as a standard following Capocasa et al. (2008), and Veberic et al. (2008). Folin–Ciocalteu reagent (1.5 mL) was added to a solution containing 0.5 mL of extract, with a known concentration (1 mg/mL). The solution was mixed and after 5 min, 1.5 mL of 7.5% sodium carbonate solution was added. The mixture was left to incubate for 90 min, and the absorbance was measured at 760 nm. The total phenolics content was calculated by a standard gallic acid graph, and the results expressed in mg of gallic acid equivalents per g (mg GAE/g) of dry weight of extract. The assay was performed in triplicate for each extract.

### Determination of total flavonoids

The total flavonoids contents in the various extracts were determined according to Djeridane et al. (2006) using a method based on the formation of a complex flavonoid-aluminum, having the maximum absorbance at 430 nm. Quercetin was used to make the calibration curve. About 1 mL of diluted sample was mixed with 1 mL of 2% aluminum trichloride (AlCl_3_) methanol solution. After incubation at room temperature for 15 min, the absorbance of the reaction mixture was measured at 430 nm UV-Vis spectrophotometer and the total flavonoid content was expressed in mg quercetin equivalent (QE) per g of extract.

### Determination of hypericin and hyperforin content

Standard solutions of hypericin 0.02 mg/mL and hyperforin 0.05 mg/mL were prepared in MeOH and stored at −18 °C. Plant extracts of various concentrations (0.02–1.00 mg/mL) were prepared by successive dilutions of a stock solution of 10 mg of dry extracts dissolved in 10 mL of methanol (Stalikas & Konidari 2001). Spectrophotometric measurements were performed on a UV-vis spectrophotometer at 590 nm for hypericin and 287 nm for hyperforin.

### Antioxidant activities

#### Free radical-scavenging activity

The evaluation of the free radical-scavenging activity was based on the measurement of the reducing ability of antioxidants toward the DPPH radical following the method described by Hatano et al. (1988) with some modifications. Various concentrations of the diluted extract were mixed with 0.50 mL DPPH (0.2 mM in meOH). The mixture was then shaken and allowed to stand at room temperature in the dark. After 30 min, the decrease in absorbance was measured at 517 nm against a blank (methanol solution) using a UV-vis spectrophotometer. A mixture consisting of 0.50 mL of methanol and 0.25 mL of DPPH solution was used as the control.

The capability to scavenge the DPPH radical was calculated using the following equation: RSA (%) = 100 × (A blank – A sample)/A blank; where A blank is the absorbance of the control reaction (containing all reagents except the test compound) and A sample is the absorbance of the test compound. Trolox was used as the positive control. Three replicates of sample were recorded. The results are expressed as Trolox equivalents (mg TEs/g).

#### Ferric reducing antioxidant power assay (FRAP)

The FRAP assay was adapted from Gardeli et al. (2008). The FRAP reagent was freshly prepared by mixing TPTZ solution (10 mM TPTZ in 40 mM HCl), ferric solution (FeCl_3_·6H_2_O, 20 mM) and acetate buffer (300 mM, pH 3.6) in proportions of 1:1:10 (v/v). To perform the assay, 900 μL of FRAP working reagent were mixed with 90 μL distilled water. 30 μL of diluted oil were then added and incubated at 37 °C in a water bath for 30 min. Absorbance at 593 nm was determined against distilled water blank. Ferrous sulfate heptahydrate solutions (100–2000 μM) were used for calibration. The FRAP values were expressed as mmol of Fe^2+^/g DW. All assays were determined in triplicate.

#### Free radical-scavenging ability by the use of a stable ABTS radical cation

The method is based on the ability of antioxidant molecules to quench the long-lived 2,20-azino-bis(3-ethylbenzothiazoline-6-sulphonic acid) radical cation (ABTS^+^), compared with that of Trolox (6-hydroxy-2,5,7,8-tetramethylchroman-2-carboxylic acid). A stable stock solution of ABTS^+ ^was produced by reacting a 7 mmol aqueous solution of ABTS with 2.45 mmol ammonium persulfate (final concentration), and allowing the mixture to stand in the dark at room temperature for 12–16 h before use (Re et al. 1999). At the beginning of the analysis day, an ABTS^+ ^working solution was obtained by diluting the stock solution with ethanol, to an absorbance of 0.70 ± 0.02 at 734 nm, verified with a UV-vis spectrophotometer. *Hypericum* aerial parts were centrifuged at 2800 *g* for 10 min and the supernatant was collected for analysis. Supernatant (10 μL) was mixed with the working solution, to a final volume of 1 mL. The inhibition percentage I(%) of radical scavenging activity was calculated as: I(%) = 100 × (A0 − AS)/A0, where A0 is the absorbance of the control and AS is the absorbance of the sample after 4 min of incubation. Trolox (in the range from 1.0 to 50 μM) was used as a reference standard. TEAC were expressed as μM of Trolox equivalents (TE), referring to 1 mL of extract.

#### Metal chelating activity

The chelating of ferrous ions by Hhu and Hper extracts was estimated as described by Dinis et al. (1994). Different concentrations of extracts were added to a 0.3 mL FeCl_2_·4H_2_O solution (1 mmol/L) and left for incubation for 5 min. The reaction was initiated by adding 0.3 mL of ferrozine (25 mmol/L). Absorbance of the solution was then measured spectrophotometrically at 562 nm. The percentage of inhibition of ferrozine-Fe^2 + ^complex formation was calculated using the formula given bellow:

Metal chelating effect (%) = (A0 − A1)/A0 × 100; where, A0 is the absorbance of the control, and A1 is the absorbance in the presence of the sample or standard. Analyses were run in triplicates.

### Enzyme inhibitory activity

Acetylcholinesterase (AChE) inhibitory activity was measured using Ellman’s method, as previously reported (Zengin et al. 2014). α-Amylase and α-glucosidase inhibitory activities were carried out by the method described by Zengin et al. (2014).

### Antimicrobial activity

#### Microbial strains

Extracts from Hhu and Hper were individually tested against a panel of microorganisms including Gram-positive bacteria and Gram-negative bacteria. Micro-organisms were provided from the culture collection of the Laboratory of Natural Substances, at National Institute of Research and Physico-chemical Analysis. The Gram-positive bacteria were *Staphylococus aureus* (strain ATCC 6538), *Staphylococcus epidermidis* (strain ATCC 12228), *Bacillus subtilis* (an environmental isolate), *Enterococcus faecium* (strain ATCC 19434), and the Gram-negative bacteria were *Escherichia coli* (strain ATCC 8739), *Salmonella typhimurium* (strain ATCC 14028), *Pseudomonas aeruginosa* (strain NCTC 10418), and *Pseudomonas aeruginosa* (strain ATCC 27853) and yeast *Candida albicans*.

#### Disc diffusion method

A suspension of the tested microorganisms was spread on the solid Mueller–Hinton media plates and incubated overnight at 37 °C. After 1 day, 4–5 loops of pure culture were transferred to saline solution in a test tube for each bacterial strain. Sterile cotton dipped into the bacterial suspension and 0.5 mL from each strand was transferred into Mueller–Hinton (M-H) agar plate. On a MHA plate were inoculated with a bacterial suspension of 0.5 McFarland turbidity standards at a density of 10^8^ cells/mL. The MeOH extracts were dissolved in 10% aqueous dimethylsulfoxide (DMSO) equivalent to 200 μg/mL, sterilized by filtering through a 0.22 μM filter and sterile paper discs and placed onto inoculated plates. The inoculum for each strain was prepared from broth cultures. Ampicillin (10 μg/disc) and Gentamicin (10 μg/disc) (USP grade, BIOMATIK, Germany) were used as positive standards in order to control the sensitivity of the microorganisms. Antibiotic disc was used for each plate and run in duplicate. Inoculated plates with discs were placed in a 37 °C incubator. After 24 h of incubation, the results were recorded by measuring the zones of growth inhibition surrounding the disc. Clear inhibition zones around the discs indicated the presence of antimicrobial activity.

#### Determination of minimum inhibitory (MIC) concentration

The MIC was determined using microdilution method recommended by Clinical and Laboratory Standards Institute (NCCLS 2009a, 2009b). The inoculum was prepared using a 24 h culture adjusted by reference to the McFarland standard and further diluted with sterile physiological saline solution to achieve approximately 10^6^ CFU/mL. A sterile 96-well microplates was used for each strain and set up as follows: in wells in raw A to G were placed 100 μL of the oil dilution added to 5 μL of the inoculum and 95 μL of the sterile MH broth. These wells are duplicated for each extract. A positive control (containing inoculum but no extract) and negative control (containing extract but no inoculum) were included on each microplate. The contents of the wells were mixed and the microplates were incubated at 37 °C for 24 h. After incubation, a 10 μL of a 5 mg/mL MTT solution, the viability of bacteria is determined by the MTT assay. In fact, MTT staining method is selected as the color with the chemical reaction of the reduction of the salt (bromide 3 (4,5-dimethylthiazol-2-yl)-2,5-diphenyltetrazolium bromide), which is yellow in blue crystals of formazan blue, were added to each well and the plates were left stirring for 30 min at room temperature.

For MBC determination, the contents of the wells showing growth inhibition were streaked onto the surface of nutrient agar plates and incubated at 37 °C for 24 h. After the incubation, the plates were observed for growth.

The MBC was estimated as the least concentration of the extract where no visible growth was observed. The MIC was defined as the lowest concentration of the total essential oil at which the microorganism does not demonstrate visible growth (Okeke et al. 2001).

### Statistical analysis

All assays were run in triplicate. The results are reported as mean values of three analyses along with the standard deviation. Data were subjected to statistical analysis using the SPSS programme, release 11.0 for Windows (SPSS, Chicago, IL). The one-way analysis of variance (ANOVA) followed by Duncan’s multiple range test were employed to study the differences between means. Values of *p* < 0.05 and *p* < 0.01 were considered statistically significant and highly significant, respectively.

## Results and discussion

### Chemical composition of *H. humifusum* and *H. perfoliatum* extracts

The mean yields ranged from 6.9% to 11.3% for *H. perfoliatum* and *H. humifusum*, respectively. Yields were then estimated on the basis of the dry weight of plant material.

The pharmaceutical activity of *H*. *humifusum* and *H*. *perfoliatum*, is attributed mainly to hypericin and hyperforin (Skalkos et al. 2005). Table 1 summarizes the results from the quantitative determination of the two major components of the extracts and their respective total phenolic and flavonoid contents. The PC value of *H. humifusum* (113.52 ± 4 g gallic acid equivalents/g DW) was statistically higher (one-way ANOVA and Duncan’s tests, *p* < 0.001) than that of *H. perfoliatum* (52.46 mg gallic acid equivalents/g DW). The TF content of *H. humifusum* (41.06 mg RE/g DW) is statistically higher than that of *H. perfoliatum* (Table 1). Results of hypericin and hyperforin content are in accordance with those obtained in TP and TF determination. The highest concentration of hypericin and hyperforin was observed in *H*. *humifusum* (90, and 30 mg/g DW, respectively) when compared to that of *H. perfoliatum* (60 and 8.5 mg/g DW, respectively).

**Table 1.** Phenolic content and concentrations of hypericin and hyperforin in *H. humifusum* and *H. perfoliatum* extracts.

	Hypericin (mg/g of dry extract)	Hyperforin (mg/g of dry extract)	TPC^b^ [mg GAE/g]	TF^c^ [mg RE/g]
*Hypericum humifusum*	90 ± 1^a^	30 ± 0.09^a^	113.52 ± 4^a^	41.06 ± 0.8^a^
*Hypericum perfoliatum*	60 ± 0.9^b^	8.5 ± 0.2^b^	48.47 ± 0.2^b^	8.45 ± 0.1^b^

Different superscript letters (a and b) in the same column indicate significant difference (*p*  < 0.05).

TPC: total phenolic compounds; TF: total flavonoids.

a Results are reported as mean ± standard deviation of three replicates (95% confidence).

b mg gallic acid equivalents/g dry weight.

c mg quercetin equivalents/g dry weight, d: mg.

### Antioxidant activities

The antioxidant capacities of *H. humifusim* and *H. perfoliatum* extracts were assayed with forth different assays in thus study: free radical scavenging (DPPH and ABTS), reducing power FRAP and metal chelating assays.

Free radical scavenging capacities were measured using DPPH radical and ABTS radical cation. The results are expressed as Trolox equivalents (mg TEs/g) and are given in Table 2. *Hypericum humifusum* with 84.63 μmoles Trolox for DPPH and 180.87 mg TEs/g for ABTS exerted greater activity than that of *H. perfoliatum* in both assays (Table 2). This potentiality is mostly correlated to the type of phenolic compounds according to species (Tawaha et al. 2010).

**Table 2.** Radical scavenging, reducing power and metal chelating activities of MeOH extracts.

	*H. humifusum*	*H. perfoliatum*
Radical scavenging activity		
DPPH^b^ [μmoles Trolox/g]	84.63 ± 1.62	52.31 ± 1.3
ABTS^b^ radical cation (mg TEs/g)	180.87 ± 1.8	80.6 ± 1.6
Reducing power		
FRAP^b^ [mmol Fe^2+^/g]	80.23 ± 0.07	50 ± 0.07
Metal chelating		
Ferrous ion chelating (mg EDTAEs/g)	3.7 ± 0.08	8.76 ± 0.08

a Results are reported as mean ± standard deviation of three replicates (95% confidence).

b μM Trolox equivalents (TE).

The reducing power may serve as significant indicator of its potential antioxidant activity. For these reason, ferric reducing power was examined (Table 2). Consistent with DPPH and ABTS radical scavenging capacity, reducing power of Hhu showed significantly higher than Hper. From these results, it seems to be hypercin and hyperforin are excellent reductants in these assays.

The chelating effects of ferrous ions were estimated. In this assay, the MeOH extracts interfered with the formation of Fe^2+ ^and ferrozine complex, thus they have chelating activity and can capture Fe^2+ ^before ferrozine. As shown in Table 2, Hper (8.76 mg EDTAEs/g), in contrast to other assays, recorded higher metal chelating activity than that of Hhu (3.7 mg EDTAEs/g). Interestingly, though Hper did not have high free radical scavenging and reducing capacities, it exhibited the superior activity in metal chelating assays. This can be explained by the fact that Hper has high concentration of other molecules with high metal chelating activity. These results are in good agreement with Nithiyanantham et al. (2012) who reported that the metal chelating capacity of phytochemicals depend on hydroxyl substitution in the structure.

### Enzyme inhibitory activity

Cholinesterase inhibitors serve as a strategy for the treatment of Alzheimer’s diseases, which is a neurodegenerative disorder. They promote an increase in the level of acetylcholine in neuronal synaptic area (Mukherjee et al. 2007). In this direction, the search for novel and safe acetylcholinesterase inhibitors from natural resources with better properties is necessary for the treatment of Alzheimer’s disease. The MeOH extracts showed a similar action on AChE (4.57 mg for Hhu and 3.86 mg GALAEs/g for Hper, respectively) (Table 3). Previously, some authors reported that *Hypericum* species were recommended for the treatment of Alzheimer’s diseases (Hofrichter et al. 2013). In these publications, the cholinesterase inhibitory property was associated with the main components of *Hypericum* species. However, inhibitory activity of Hhu and Hper on AChE may be due to the high level of hypericin.

**Table 3.** Enzyme inhibitory activity of the MeOH extracts (mean ± SD).

Samples	Acetyl cholinesterase (mg GALAEs/g)	α-Amylase (mmol ACEs/g)	α-Glucosidase (mmol ACEs/g)
*Hypericum humifusum*	4.57 ± 0.3	2.55 ± 0.08	8 ± 0.08
*Hypericum perfoliatum*	3.86 ± 0.02	0.73 ± 0.04	3.88 ± 0.07

GALAEs: galanthamine equivalents; ACEs: acarbose equivalents.

Diabetes mellitus is one of the major public health problems. It is characterized by chronic hyperglycemia with disturbance of glucose metabolism resulting from the defect in insulin secretion (Dong et al. 2012). α-Amylase and α-glucosidase are key enzymes in hydrolysis of starch and oligosaccharide (Gray 1995). The inhibition of these enzymes is an important strategy in the management blood glucose level. It is important to evaluate the anti-diabetic properties of medicinal plants and their products to develop natural compounds with low toxicity. Antidiabetic activity was investigated by α-amylase and α-glucosidase inhibitory assays. As shown in Table 3, the highest α-glucosidase inhibitory activity was recorded in the Hhu with 8 mmol ACEs/g. in the same way α-amylase inhibitory activity of Hhu (2.55 mmol ACEs/g) was higher than that of Hper (0.73 mmol ACEs/g). The anti-diabetic activity of the extracts is related to main components. Gulam et al. (2009) demonstrated that hyperforin is responsible for the antidiabetic activity.

### Antimicrobial activity

The antimicrobial activity of extracts was examined by disc diffusion and microdilution susceptibility assay against eight bacterial and one yeast strains.

The extracts exhibited variable MICs and important antimicrobial activity, depending on the microbial strains (Table 4). *Hypericum humifusum* showed very strong activity against *E. coli*, *Pseudomona* sp. and *Bacillus subtilis* (200-250 μg mL^−1^). The highest MIC for *Salmonella typhimurium* was 500 μg/mL, whereas the lowest MIC was 200 μg/mL for *Bacillus subtilis*. Mukherjee et al. (2003) reported that the leaf of *H. mysorense* and *H. patulum* have a very broad spectrum against panel of microorganisms.

**Table 4.** MIC and MBC results of of *H. humifusum* and *H. perfoliatum* extracts against selected bacterial and yeast strains.

	*CON+*		*H. humifusum*		*H. perfoliatum*	
Bacterial strain	MIC	MBC	MIC	MBC	MIC	MBC
*Escherichia coli* G(−)	18.5	25	350	500	350	600
*Salmonella typhimurium* G(−)	15	24	500	550	500	650
*Pseudomonas aeruginosa* G(−)	1.6	3.5	300	600	350	650
*Pseudomonas aeruginosa* G(−)	1	2.5	300	600	350	600
*Enterococcus faecium* G(+)	10.5	13	250	400	450	500
*Staphylococus aureus* G(+)	5	10.5	250	400	300	400
*Staphylococcus epidermidis* G(+)	6.5	12	200	350	350	450
*Bacillus subtilis* G(+)	15	30	350	350	350	450
*Candida albicans*	5	11.5	300	350	250	350

CON+: positive control, antibiotic: Ampicillin for all strains except for *Pseudomonas aeruginosa* where positive control and Gentamicin.

MIC: minimal inhibitory concentration (μg mL^−1^).

MIC was defined as the lowest concentration of the compounds produced >95% growth reduction compared with the growth in the control well.

MBC: minimum bactericidal concentration (μg mL^−1^).

Extracts from the two species showed a very high bactericidal effect. However, both Hhu (MBC = 500–600 μg/mL) and Hper (MBC = 600–650 μg/mL) showed a high-bactericidal activity against the Gram-negative bacteria *E. coli*, *S. typhimurium* and *P. aeruginosa*. The lowest MBC values have been detected against the two Gram-positive bacteria *S. epidermidis* and *B. subtilis* for Hhu and *Candida albicans* for Hper.

*Hypericum* genus has been found to yield compounds having antimicrobial property. Inhibitory effects of *H. perforatum* on Gram-positive bacteria have also been observed and attributed to hyperforin, the main acylphloroglucinol isolated from this plant. Phloroglucinol derivatives, found frequently in the lipophilic (Avato et al. 2004) fractions of several *Hypericum* species, have demonstrated antibacterial activities against microorganisms such as *S. aureus*, *B. cereus*, *B. subtilis* and *Nocardia gardenen*. The *Staphylococcus* species have been identified as the leading bacteria which are mostly studied in terms of their interaction with this genus. *Escherichia coli*, *Mycobacterium* sp., *Staphylococcus* sp., *Streptococcus* sp. have been commonly used for determination of antimicrobial effect of *H. humifusum* (Nogueira et al. 2013). Gibbons et al. (2002) showed the impact of the chloroform and methanol extracts of 34 different species of *Hypericum* on *Staphylococcus*. According to the results, it has been emphasized that *Hypericum* genus is of a great potential for anti-staphylococcal effect.

## Conclusions

*Hypericum* species have become a popular area for many scientific researches. It has been estimated that it would be a significant material for both biological and chemical studies mainly for its availability and widespread distribution. In this study, a large number of chemical studies on *H. humifusum* and *H. perfoliatum* have been reviewed. Both species showed a good phenolic, flavonoid, hypericin and hyperforin content which explain their high-antioxidant capacities as evaluated by four *in vitro* assays. However, the first species seems to be more important as a source of these natural compounds. It also showed a better potential biological activity as enzyme inhibitors and antimicrobial agents.
